# Melatonin Regulates Osteoblast Differentiation through the m6A Reader hnRNPA2B1 under Simulated Microgravity

**DOI:** 10.3390/cimb46090572

**Published:** 2024-09-01

**Authors:** Quan Sun, Liqun Xu, Zebing Hu, Jingchun Liu, Tingfei Yu, Meng Li, Shu Zhang, Fei Shi

**Affiliations:** 1 The Key Laboratory of Aerospace Medicine, Ministry of Education, Air Force Medical University, Xi’an 710032, China; sunquan@fmmu.edu.cn (Q.S.); aliqunxu@fmmu.edu.cn (L.X.); zebinghu@fmmu.edu.cn (Z.H.); limeng981030@163.com (M.L.); 2 No. 5 Cadet Regiment, School of Basic Medical Sciences, Air Force Medical University, Xi’an 710032, China; 15538367994@163.com (J.L.); 18876087300@163.com (T.Y.)

**Keywords:** simulated microgravity, melatonin, hnRNPA2B1, MC3T3-E1 cells, osteoblast differentiation

## Abstract

Recent studies have confirmed that melatonin and N6-methyladenosine (m6A) modification can influence bone cell differentiation and bone formation. Melatonin can also regulate a variety of biological processes through m6A modification. Heterogeneous nuclear ribonucleoprotein A2/B1 (hnRNPA2B1) serves as a reader of m6A modification. In this study, we used the hindlimb unloading model as an animal model of bone loss induced by simulated microgravity and used 2D clinorotation to simulate a microgravity environment for cells on the ground. We found that hnRNPA2B1 was downregulated both in vitro and in vivo during simulated microgravity. Further investigations showed that hnRNPA2B1 could promote osteoblast differentiation and that overexpression of hnRNPA2B1 attenuated the suppression of osteoblast differentiation induced by simulated microgravity. We also discovered that melatonin could promote the expression of hnRNPA2B1 under simulated microgravity. Moreover, we found that promotion of osteoblast differentiation by melatonin was partially dependent on hnRNPA2B1. Therefore, this research revealed, for the first time, the role of the melatonin/hnRNPA2B1 axis in osteoblast differentiation under simulated microgravity. Targeting this axis may be a potential protective strategy against microgravity-induced bone loss and osteoporosis.

## 1. Introduction

Bones are dynamic organs that maintain a balance between osteoclast-mediated bone resorption and osteoblast-mediated bone formation. This process is regulated by many factors, including inflammation, nutrition, hormones, and mechanical stress [1]. The mechanical unloading of long-duration spaceflight disrupts the balance of bone remodeling, reducing bone formation, leading to disuse osteoporosis, and increasing the risk of fracture, which are serious threats to the health of astronauts [2,3,4]. Prolonged exposure to microgravity causes astronauts to lose 1–2% of their bone mass per month, while post-menopausal women lose 1% of their bone mass per year [5]. However, the specific mechanism by which microgravity affects bone homeostasis remains unclear. It has been shown that when osteoblasts and osteoclasts are co-cultured in vitro in microgravity, Wnt signaling is downregulated, osteoclastic markers are increased, and osteoblastic markers are decreased [6]. Osteoblast proliferation and metabolism have been found to be inhibited in microgravity [7]. The internal morphology of osteoblasts is susceptible to alterations in microgravity, as evidenced by notable changes in the shape and size of osteoblast nuclei in microgravity [8]. Furthermore, osteoblasts undergo programmed cell death in microgravity, which effectively reduces the number of osteoclasts [9,10]. The secretion of chemokines by osteoblasts is increased in microgravity, thereby promoting the recruitment of osteoclasts and stimulating the process of bone resorption [11]. The expression of proteins associated with cell differentiation and maturation is reduced, including alkaline phosphatase (ALP), osteocalcin (OCN), and type I collagen (Col1a1) [12]. Weakened osteoblast differentiation has been reported to play a key role in unloading-induced bone loss [13,14,15]. However, there is currently no safe and effective protective measure against this process, and it is necessary to investigate potential molecular mechanisms to provide a scientific basis for formulating more effective interventions.

Melatonin (N-acetyl-5-methoxytryptamine, MT) is an indole-like hormone secreted by the pineal glands of vertebrates. Melatonin influences the circadian rhythm, regulates the sleep–wake cycle, inhibits the growth of tumors, regulates the immune system, and is an antioxidant [16]. In addition, melatonin plays a positive role in maintaining bone homeostasis. Melatonin has been demonstrated to induce osteogenic differentiation of bone marrow mesenchymal stem cells (BMSCs) and to promote osteoblast differentiation while simultaneously inhibiting osteoclastogenic activation [17,18,19,20]. Melatonin receptors (MT1 and MT2) belong to the family of G protein-coupled receptors [21]. Melatonin is able to regulate a wide range of intracellular signaling cascades and lead to the activation of various ion channels via its receptors. Multiple molecular pathways have been reported for melatonin regulation of osteoblast differentiation. Studies have shown that melatonin can induce osteoblast differentiation by affecting the PI3K/Akt, PKD/p38, BMP/Smad, NF-κB, and Wnt/β-catenin pathways and by increasing the expression of osteogenic transcription factors, such as Runx2, Col1a1, OCN, and Osterix (OSX) [16,22,23,24,25,26]. Melatonin has the potential to maintain bone homeostasis and prevent osteoporosis without adverse effects [27,28,29,30]. Previous studies suggest that melatonin may become a novel drug used to prevent unloading-induced bone loss [31,32]. However, the specific molecular mechanisms by which melatonin regulates bone metabolism in microgravity remain to be fully elucidated. Furthermore, melatonin may act via N6-methyladenosine (m6A) [33,34].

M6A is the most common epigenetic RNA modification in eukaryotes [35]. It can precisely regulate the expression of genes involved in bone metabolism and affects the functions of osteoblasts, bone marrow mesenchymal stem cells, and osteoclasts. It is a potential target for promoting bone formation and preventing osteoporosis [36,37,38]. M6A recognition proteins, also known as “readers”, can specifically bind to m6A reading sites, thereby affecting RNA shearing, translation, degradation, and other processes [39,40]. Heterogeneous nuclear ribonucleoprotein A2/B1 (hnRNPA2B1) is an m6A reader that is involved in regulating nuclear mRNA maturation, transport, and metabolism, as well as promoting the processing and splicing of primary microRNAs (miRNAs) [41]. However, whether hnRNPA2B1 regulates osteoblasts under simulated microgravity has not been investigated.

In this study, we observed that hnRNPA2B1 was downregulated under simulated microgravity. We found that hnRNPA2B1 promoted osteoblast differentiation and could partially reverse the inhibition of osteoblast differentiation caused by simulated microgravity. We also discovered that melatonin could promote the expression of hnRNPA2B1 under simulated microgravity and that the promotion of osteoblast differentiation by melatonin was partially dependent on hnRNPA2B1. Our studies revealed, for the first time, the role of the melatonin/hnRNPA2B1 axis in osteoblast differentiation under simulated microgravity. Targeting this axis may be a potential protective strategy against microgravity-induced bone loss and osteoporosis.

## 2. Materials and Methods

### 2.1. Animals and Experimental Design

C57BL/6J mice (6-month-old males) were obtained from the Animal Centre of Air Force Medical University (Xi’an, China). All protocols were approved by the Experimental Safety Committee and the Animal Care Committee of Air Force Medical University. The mice were randomly numbered with different ear tags, with each tag corresponding to a random number. Subsequently, the mice were separated into four groups according to the random numbers’ order (five mice per group): control (Con), hindlimb unloading (HLU), HLU supplemented with normal saline (HLU + NS), and HLU supplemented with melatonin (HLU + MT). The HLU model is a well-accepted animal model of bone loss induced by simulated microgravity. The male C57BL/6J mice used in the HLU model were maintained under standard conditions (22 °C and 12 h light/12 h dark cycle). The mice were suspended by their tails for 21 days with the long axes of their bodies held off the ground at 30° angles so that their hindlimbs were suspended. The mice’s forelimbs touched the floor, allowing them to move freely within their cages and to access food and water without difficulty. The control mice were housed in the same cage environment without the tail-suspension device. The hindlimb unloading procedure is shown in Figure S1.

Melatonin (Sigma-Aldrich, St. Louis, MO, USA) was dissolved in ethanol, and then normal saline was added to prepare a solution for intraperitoneal injection (final ethanol concentration: 2%). The HLU + MT group received intraperitoneal injections containing 20 mg/kg of melatonin. The injections were given on alternate days at 8 p.m. for 21 days. After 3 weeks of tail suspension, the mice were euthanized with CO_2_. Blood samples were collected in a centrifuge tube with an anticoagulant, and the plasma was isolated and examined using ELISA. Bilateral femurs and tibias were then removed from the mice. Immunohistochemistry (IHC) and micro-CT scanning were used for further analysis.

### 2.2. Micro-CT Scanning

The femurs were fixed with 4% paraformaldehyde and subsequently scanned using a micro-CT system (performed by PerkinElmer, Waltham, MA, USA). The scan parameters were set as follows: current of 80 μA; voltage of 50 kV; 360° rotational scan; angle gain of 0.5°; 10.0 μm spatial resolution; 5.12 mm total thickness of scanned area; scanning time for each sample of about 14 min. A region of interest (ROI) measuring 2.5 × 2.5 × 3 mm^3^ was selected approximately 1.5 mm away from the growth plate for the detection of bone mass and microstructure, followed by 3D reconstruction. Analyze 12.0 Properties software was used for 3D reconstruction and analysis of ROI. The following parameters of ROI were quantified: bone mineral density (BMD); relative bone volume (BV/TV); trabecular number (Tb. N); bone trabecular separation (Tb. Sp); cortical bone thickness (Ct. Th); cortical bone area (Ct. Ar); total cortical bone area (Tt. Ar).

### 2.3. Immunohistochemistry (IHC)

Tissue sections were soaked in xylene for 10 min, and then the xylene liquid was replaced, and they were soaked for a further 10 min to dissolve the paraffin in the sections and facilitate subsequent staining. The dewaxed sections were placed in anhydrous ethanol, 95% ethanol, 85% ethanol, and 70% ethanol for 5 min to complete the hydration process. The hydrated paraffin slides were placed in a freshly prepared citrate buffer, heated and boiled for 10 min, cooled to room temperature, heated and boiled again at low heat for 10 min, and cooled to room temperature. Then, the antigen site was fully exposed. The slides were rinsed with PBS for 5 min three times, soaked in 3% H_2_O_2_ for 10 min, and rinsed with PBS three times for 3 min each. The slides were dried with filter paper and incubated with 3% goat serum in a wet box for 30 min at room temperature. Then, we discarded the goat serum and added an hnRNPA2B1 antibody (1:1000, Abclonal, Wuhan, China) solution. The slides were incubated overnight at 4 °C. Then, we rinsed the slides with PBS for 5 min 3 times. We dried the slides with filter paper, added a secondary antibody solution, and incubated the slides at room temperature for 50 min. Then, we rinsed the slides with PBS for 5 min 3 times. A DAB color development solution was added. We observed the slides using a microscope and washed them with water to stop color development. We soaked them in a hematoxylin solution for 3 min, and then rinsed them with distilled water. We used 1% hydrochloric acid–ethanol differentiation and, further rinsed the slides with distilled water. Then, we used 70% ethanol, 85% ethanol, anhydrous ethanol, and xylene to dehydrate the slides for 5 min. We then dried the sections and sealed them with neutral resin.

### 2.4. Enzyme-Linked Immunosorbent Assay (ELISA)

Each blood sample was centrifuged at 3000 rpm and 4 °C for 15 min and the plasma supernatant was removed and placed in a fresh centrifuge tube. The levels of melatonin were quantified using the ELISA kits, in accordance with the manufacturer’s instructions. The absorbance at 450 nm was measured using a microplate reader (S/N 415-2687, Omega Bio-Tek, Ortenberg, Germany).

### 2.5. Cell Culture and In Vitro Differentiation

MC3T3-E1 cells (Shanghai Cell Bank, Chinese Academy of Sciences, China) were routinely cultured in an α-MEM complete medium (Hyclone, Logan, UT, USA) containing 100 mL/L fetal bovine serum and 10 mL/L penicillin/streptomycin (Gibco, Big Island, NY, USA) in an incubator at 37 °C (5% CO_2_ and 95% humidity). When investigating pre-osteoblast differentiation, we used an osteogenic induction medium (α-MEM complete medium, 100 nmol/L dexamethasone, 50 µg/mL vitamin C, and 10 mmol/L sodium β-glycerophosphate) for an osteogenic induction culture. Four to eight generations of cells were selected for this study.

### 2.6. Two-Dimensional Clinorotation

Two-dimensional clinorotation (developed by the China Astronaut Research and Training Centre, Beijing, China) is a widely accepted method for simulating microgravity in vitro on the ground. MC3T3-E1 cells were routinely cultured in 25 cm^2^ customized rotary flasks with an inoculation density of 5 × 10^5^ cells/flask. Two-dimensional (2D) clinorotation drove the customized flasks to rotate at a specific speed, thereby simulating a microgravity environment at the cellular level. After cell attachment, we filled the culture flasks with normal or osteogenic induction media and carefully removed any air bubbles. The flasks in the simulated microgravity group (Clino group) were then fixed in a 2D clinostat, set at 24 rpm, and rotated at a constant temperature of 37 °C for 24 h, 48 h, or 72 h. The control group (Con group) was placed in the same environment without the 2D clinostat treatment and cultured routinely for the same durations.

### 2.7. Cell Transfection

A Lipofectamine 2000 kit (Invitrogen, Waltham, MA, USA) was employed for cell transfection. siRNA targeting hnRNPA2B1 (80 nM) and the negative control were purchased from GenePharma (Shanghai, China) and used for transfection. The cells were transfected at a density of 30–50% confluence in accordance with the manufacturer’s instructions. pEX-hnRNPA2B1 plasmids (200 ng/μL) (Genepharma, Shanghai, China) and the negative control were used to transfect osteoblasts in order to induce overexpression. The sequences of the siRNAs and negative controls are shown in Table 1.

### 2.8. Quantitative Real-Time PCR (qRT-PCR) and Analysis

The total RNA was extracted from the cells using the RNAiso Plus reagent (TaKaRa, Tokyo, Japan). A Prime Script™ RT Master Mix reagent kit (TaKaRa, Tokyo, Japan) was used to convert the mRNA into cDNA. Target gene expression was quantified using a CFX96 real-time PCR detection system (BIO-RAD, Hercules, CA, USA) with SYBR Premix Ex Taq TM II (TaKaRa, Tokyo, Japan), with GAPDH serving as an endogenous control. The mRNA level was normalized to the GAPDH mRNA level using the 2^−ΔΔCT^ method. All primers are listed in Table 2.

### 2.9. Western Blotting and Analysis

MC3T3-E1 cells were collected via trypsin digestion, and the total protein was extracted using M-PER Mammalian Protein Extraction Reagent (Thermo Scientific, Waltham, MA, USA) containing a 100 mL/L protease inhibitor (Roche, Basel, Switzerland). The protein samples were ultrasonically lysed and centrifuged at 12,000 rpm for 10 min at 4 °C. The supernatant was aspirated, and the aspirated volume was recorded. The concentration of each protein sample was determined using a bicinchoninic acid (BCA) protein assay kit (Thermo Scientific, Waltham, MA, USA). A loading buffer was then added to the protein samples. Equal amounts of the protein samples were added to NuPAGE™ Bis-Tris Protein Gels (Invitrogen, Carlsbad, CA, USA) and subjected to electrophoresis for a period of two hours. The proteins were then transferred to polyvinylidene difluoride membranes, which were maintained in an ice bath throughout this process. The membranes were then immersed in 5% skimmed milk for a period of four hours at room temperature. The membranes were then incubated with primary antibodies overnight at a temperature of 4 °C. The following primary antibodies were used: GAPDH (1:50,000, 60004-1-Ig; Proteintech, Chicago, IL, USA), Runx2 (1:1000, #12556S; Cell Signaling Technology, Danvers, Massachusetts, USA), Col1al (1:1000, A1352; Abclonal, Wuhan, China), and hnRNPA2B1 (1:1000, A21802; Abclonal, Wuhan, China). After washing for one hour, the membranes were incubated with either an HRP-conjugated goat anti-mouse antibody (1:5000, ZB-2305; ZSGB-BIO, Beijing, China) or an anti-rabbit secondary antibody (1:5000, ZB-2301; ZSGB-BIO, Beijing, China) for one hour and developed with a Super Signal West substrate (Thermo Fisher Scientific, Waltham, MA, USA). Densitometric analysis of the bands was performed using Image J 1.8.0 software.

### 2.10. Alkaline Phosphatase Staining (ALP Staining)

Alkaline phosphatase staining was performed using a BCIP/NBT staining kit (Beyotime Biotechnology, Shanghai, China). The 6-well plates in which the cells had been cultured were rinsed with PBS and allowed to dry. They were then fixed with 4% paraformaldehyde for 15 min and rinsed with PBS for 5 min three times. After controlled drying, the BCIP/NBT staining solution was added to each well and incubated for 30 min at room temperature in the dark. Subsequently, the working solution was removed at the end of the chromatography, and the chromatography reaction was terminated via two washes with ddH2O. After controlled drying, digital images were taken using a camera.

### 2.11. ALP Activity Assay

M-PER Mammalian Protein Extraction Reagent (Thermo Fisher Scientific, USA) was used to isolate the total protein from MC3T3-E1 cells. The protein concentration was determined using a Pierce™ BCA Protein Assay Kit (Thermo Fisher Scientific, USA). The ALP activity was assessed using an ALP assay kit (Nanjing Jiancheng Technological, Nanjing, China). The amount of phenol produced after 1 g of protein reacted with the substrate for 15 min at 37 °C was applied to evaluate the ALP activity (IU/L).

### 2.12. Rescue Experiments

MC3T3-E1 cells were cultured in rotary flasks, and transfection was performed when the cell density reached approximately 60%. Following a period of 6–8 h, the culture flasks were filled with medium and sealed after all the air bubbles were expelled. The flasks were then placed in a 2D clinostat for a period of 48 h. The cells were divided into four groups: the Con group, the Clino group, the Clino + pEX group, and the Clino + pEX-hnRNPA2B1 group.

### 2.13. Statistical Analysis

All quantitative data were expressed as the means ± standard deviations from at least three experiments. Statistical analyses were conducted using GraphPad Prism 9.0 (GraphPad Software, La Jolla, CA, USA) and SPSS 22.0 software. The *t* test was adopted for comparisons between two groups. One−way ANOVA or two−way ANOVA was used for comparisons among multiple groups, followed by Tukey′s multiple-comparison test. A *p*−value of less than 0.05 was deemed to indicate a statistically significant difference. The required sample sizes for achieving significant statistical power in the present study were estimated using the G*Power software version 3.0.10 (Heinrich−Heine University, Düsseldorf, Germany) and the effect sizes obtained from the preliminary studies.

## 3. Results

### 3.1. Expression of hnRNPA2B1 in Osteoblasts under Simulated Microgravity

**Figure 1:**
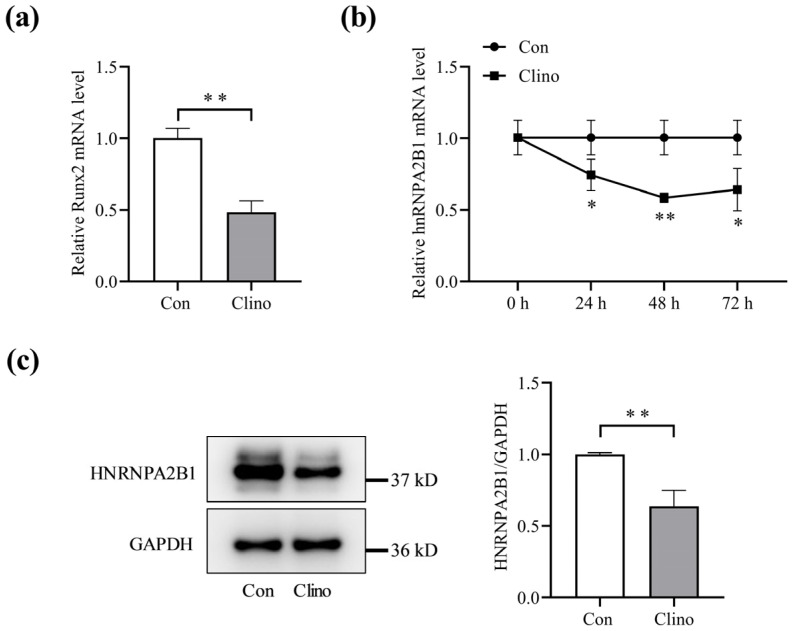
Expression level of hnRNPA2B1 in osteoblasts under simulated microgravity. (**a**) mRNA expression level of Runx2 in MC3T3-E1 cells under simulated microgravity after 48 h (N = 3). (**b**) mRNA expression level of hnRNPA2B1 in MC3T3-E1 cells under simulated microgravity for 24 h, 48 h or 72 h (N = 3). (**c**) Protein expression level of HNRNPA2B1 in MC3T3-E1 cells under simulated microgravity for 48 h (N = 3). * *p* < 0.05, ** *p* < 0.01 vs. control.

To investigate the expression and significance of hnRNPA2B1 in osteoblasts under simulated microgravity, we subjected MC3T3-E1 cells to 2D clinorotation for 48 h. Runx2 mRNA expression was significantly downregulated (Figure 1a), which is consistent with previous studies and indicates that the simulated microgravity model was successfully established. MC3T3-E1 cells were then subjected to 2D clinorotation for 24, 48, or 72 h. It was observed that the mRNA expression of hnRNPA2B1 was continuously downregulated compared to the control group and reached a nadir after 48 h under 2D clinorotation conditions (Figure 1b). The Western blotting results demonstrate a significant decrease in the protein level of hnRNPA2B1 after 48 h of 2D clinorotation (Figure 1c). These observations indicate that simulated microgravity inhibited the expression of hnRNPA2B1 in osteoblasts.

### 3.2. hnRNPA2B1 Regulates Osteoblast Differentiation

**Figure 2:**
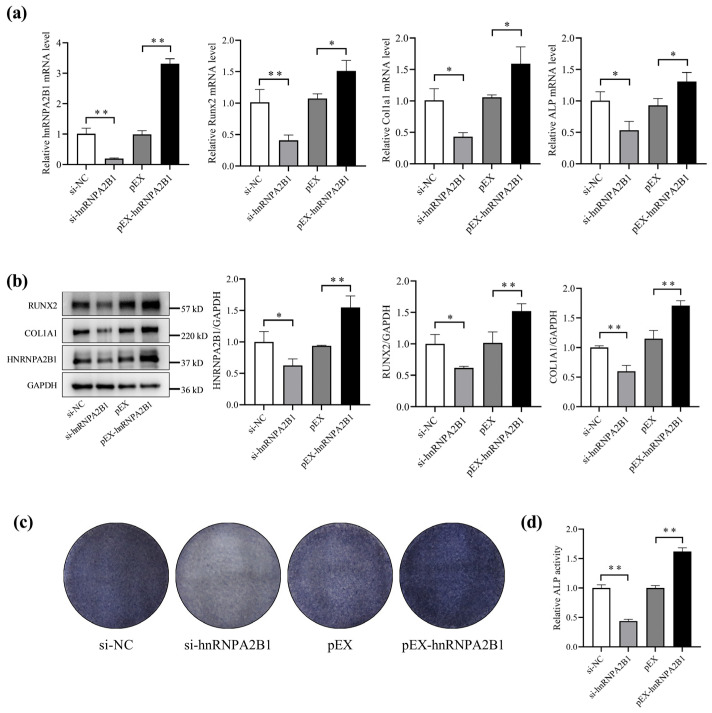
hnRNPA2B1 regulates osteoblast differentiation. (**a**) mRNA expression levels of hnRNPA2B1, Runx2, Col1a1, and ALP after knockdown or overexpression of hnRNPA2B1 (N = 3). (**b**) Protein expression levels of RUNX2, COL1A1, and HNRNPA2B1 after knockdown or overexpression of hnRNPA2B1 (N = 3). (**c**) Representative staining images of ALP in MC3T3-E1 cells (N = 3). (**d**) Analysis of ALP activity in MC3T3-E1 cells (N = 3). * *p* < 0.05, ** *p* < 0.01 vs. control.

Previous studies have shown that simulated microgravity inhibits osteoblast differentiation. We speculated that this may be associated with a reduction in hnRNPA2B1 under simulated microgravity. To investigate the impact of hnRNPA2B1 on the differentiation function of osteoblast, we transfected si-hnRNPA2B1, pEX-hnRNPA2B1, or corresponding controls into MC3T3-E1 cells. Following transient transfection, the mRNA and protein expression of hnRNPA2B1 in the MC3T3-E1 cells exhibited corresponding changes (Figure 2a,b). The overexpression of hnRNPA2B1 in the MC3T3-E1 cells resulted in significant increases in the mRNA expression levels of Runx2, Col1a1, and ALP, as well as the protein expression levels of RUNX2 and COL1A1 (Figure 2a,b). Conversely, knocking down hnRNPA2B1 led to decreases in the aforementioned mRNA and protein expression levels (Figure 2a,b). The results of the ALP staining assay and ALP activity are consistent with the above results (Figure 2c,d). These results show that hnRNPA2B1 promoted osteoblast differentiation, suggesting that the inhibition of osteoblast differentiation under simulated microgravity may be associated with reduced hnRNPA2B1.

### 3.3. Overexpression of hnRNPA2B1 Attenuates the Inhibitory Effect of Simulated Microgravity on Osteoblast Differentiation in MC3T3-E1 Cells

**Figure 3:**
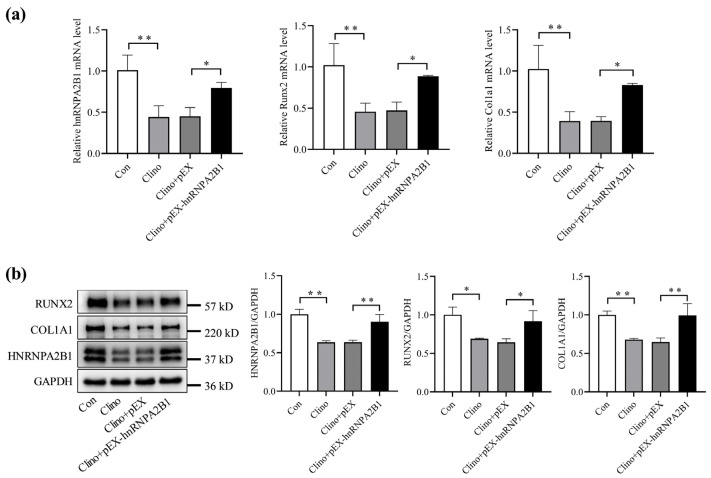
Overexpression of hnRNPA2B1 attenuates the inhibitory effect of simulated microgravity on osteoblast differentiation in MC3T3-E1 cells. (**a**) mRNA expression levels of hnRNPA2B1, Runx2, and Col1a1 in MC3T3-E1 cells after overexpression of hnRNPA2B1 under simulated microgravity for 48 h (N = 3). (**b**) Protein expression levels of HNRNPA2B1, RUNX2, and COL1A1 in MC3T3-E1 cells after overexpression of hnRNPA2B1 under simulated microgravity for 48 h (N = 3). * *p* < 0.05, ** *p* < 0.01 vs. control.

To confirm the effect of hnRNPA2B1 on osteoblast differentiation under simulated microgravity, we transfected pEX-hnRNPA2B1 and a corresponding control into MC3T3-E1 cells for 12 h. The MC3T3-E1 cells were then cultured in 2D clinorotation for 48 h to simulate microgravity in vitro. The qRT-PCR and Western blotting results show significant decreases in the mRNA and protein expression of hnRNPA2B1, Runx2, and Col1a1 in the Clino group compared to the control group (Figure 3a,b). Notably, the mRNA and protein expression levels of Runx2 and Col1a1 were significantly increased in the Clino + pEX-hnRNPA2B1 group compared to the Clino + pEX group (Figure 3a,b). These results indicate that the overexpression of hnRNPA2B1 could partially alleviate the inhibition of osteoblast differentiation caused by simulated microgravity.

### 3.4. Melatonin Promotes hnRNPA2B1 Expression in Osteoblasts under Simulated Microgravity and Improves Bone Architecture in HLU Mice

**Figure 4:**
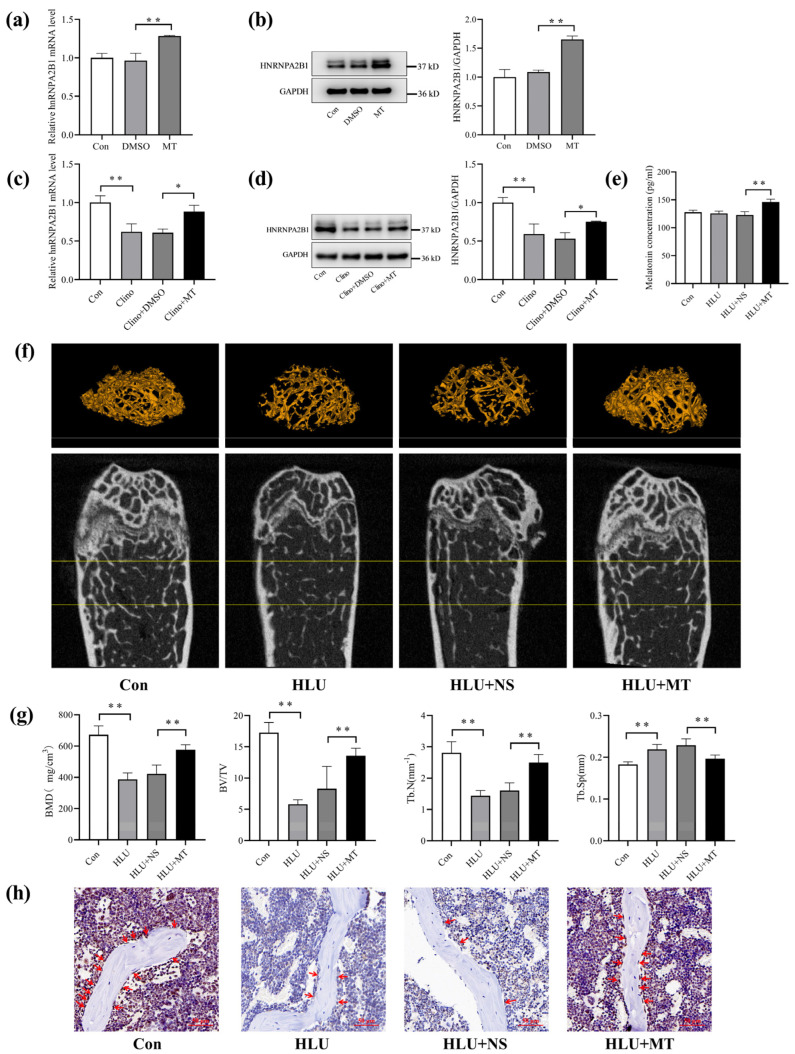
Melatonin promoted hnRNPA2B1 expression in osteoblasts under simulated microgravity and improved bone architecture in HLU mice. (**a**) mRNA expression level of hnRNPA2B1 with melatonin treatment (N = 3). (**b**) Protein expression level of HNRNPA2B1 with melatonin treatment (N = 3). (**c**) mRNA expression level of hnRNPA2B1 under simulated microgravity for 48 h with melatonin treatment (N = 3). (**d**) Protein expression level of HNRNPA2B1 under simulated microgravity for 48 h with melatonin treatment (N = 3). (**e**) Plasma melatonin concentration in mice (N = 5). (**f**) Representative images of micro−CT and three−dimensional reconstruction of the distal femurs of mice in each group (N =  5). Between the two yellow lines is the ROI area. (**g**) Three−dimensional measurement of BMD, BV/TV, Tb. N and Tb. Sp in the ROI region of the distal femurs of mice from each group (N = 5). (**h**) Representative images of hnRNPA2B1 immunohistochemical staining in the tibia of mice from each group. The red arrows indicate the number of hnRNPA2B1−positive osteoblasts on the bone−forming surfaces. Scale bar, 50 μm. * *p* < 0.05, ** *p* < 0.01 vs. control. BMD: bone mineral density; BV/TV: the ratio of bone volume to total volume; Tb. N: trabecular bone number; Tb. Sp: trabecular separation.

To investigate the effect of melatonin on hnRNPA2B1 in osteoblasts, we used dimethyl sulfoxide (DMSO) to solubilize melatonin at a DMSO/medium volume ratio of 1/2000. MC3T3-E1 cells were cultured with a medium containing 100 nmol/L melatonin. The results show that the mRNA and protein expression of hnRNPA2B1 in the MC3T3-E1 cells were upregulated by melatonin (Figure 4a,b). In order to ascertain whether melatonin affects hnRNPA2B1 expression under simulated microgravity, HLU mice and cells cultured under 2D clinorotation conditions were selected for in vivo and in vitro studies. We incubated MC3T3-E1 cells with a medium containing 100 nmol/L melatonin or a control. The MC3T3-E1 cells were then cultured in 2D clinorotation for 48 h. We found that melatonin significantly increased the expression of hnRNPA2B1 in osteoblasts under simulated microgravity (Figure 4c,d).

To establish the HLU model, we suspended the tails of C57BL/6J mice for 21 days. The HLU mice received intraperitoneal injections of a melatonin (MT) solution or normal saline (NS) every other day. The mice were divided into a CON group, an HLU group, an HLU + NS group, and an HLU + MT group. The plasma melatonin concentrations in the mice were measured using the ELISA method, and the plasma melatonin concentration in the HLU + MT group was significantly higher than that in the HLU + NS group (Figure 4e). Micro-CT was used to examine the bone mass and the microstructures of the femurs, and it revealed a remarkable reduction in bone mass in the HLU group compared to the CON group, whereas the HLU + MT group had significantly improved bone mass compared to the HLU + NS group (Figure 4f and Figure S2). Furthermore, the bone mineral density (BMD) on the trabecular area, trabecular number (Tb. N), and ratio of bone volume to total volume (BV/TV) in the HLU group were lower than those in the control group, while the declines in these parameters were reversed in the HLU + MT group (Figure 4g). In addition, bone trabecular separation (Tb. Sp) was increased in the HLU group compared to the control group, and this increase was alleviated after the melatonin treatment (Figure 4g). In addition, the cortical bone′s bone mineral density (BMD), cortical bone thickness (Ct. Th), cortical bone area (Ct. Ar), and total cortical bone area (Tt. Ar) in the HLU group were lower than those in the control group, while the declines in these parameters were reversed in the HLU + MT group (Figure S2).

Immunohistochemical staining clearly showed that the number of hnRNPA2B1−positive cells on the bone-forming surfaces was lower in the HLU group compared to the control group. In addition, the HLU + MT group exhibited a higher number of positive cells compared to the HLU + NS group (Figure 4h). These results indicate that melatonin could promote hnRNPA2B1 expression in osteoblasts under simulated microgravity and could increase bone mass and improve the trabecular microarchitecture in HLU mice.

### 3.5. Melatonin Regulates Osteoblast Differentiation by Targeting hnRNPA2B1

**Figure 5:**
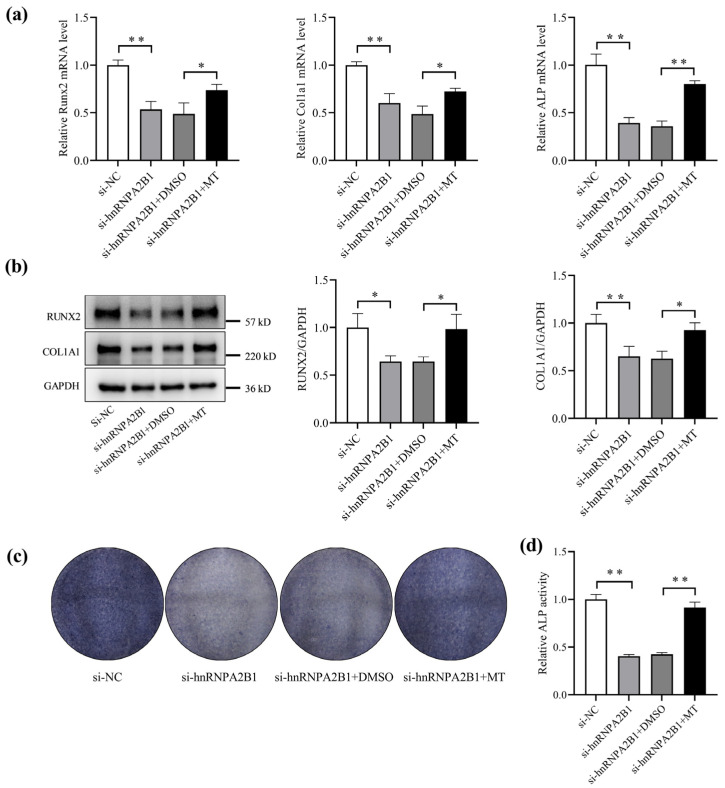
Melatonin regulates osteoblast differentiation by targeting hnRNPA2B1. (**a**) mRNA expression levels of Runx2, Col1a1, and ALP with melatonin treatment after transfection of si-hnRNPA2B1 (N = 3). (**b**) Protein expression levels of RUNX2 and COL1A1 with melatonin treatment after transfection of si-hnRNPA2B1 (N = 3). (**c**) Representative staining images of ALP in MC3T3-E1 cells (N = 3). (**d**) Analysis of ALP activity in MC3T3-E1 cells (N = 3). * *p* < 0.05, ** *p* < 0.01 vs. control.

To confirm whether the regulatory effect of melatonin on osteoblasts depends on hnRNPA2B1, MC3T3-E1 cells were transfected with si-hnRNPA2B1 and treated with melatonin. Compared to the si-hnRNPA2B1 + DMSO group, the mRNA expression levels of Runx2, Col1a1, and ALP were significantly higher in the si-hnRNPA2B1 + MT group (Figure 5a). The protein levels of Runx2 and Col1a1 were also significantly higher in the hnRNPA2B1 + MT group (Figure 5b). Furthermore, the ALP activity and ALP staining demonstrated consistent trends (Figure 5c,d). These observations collectively suggest that melatonin regulates osteoblast differentiation partly via hnRNPA2B1.

## 4. Discussion

Mechanical stimulation is critical for the maintenance of bone homeostasis, and bone loss due to mechanical unloading in microgravity has long been an important medical challenge limiting long-term space flight [42,43]. Previous studies have indicated that inhibition of osteoblast-mediated bone formation is the primary cause of bone loss during spaceflight [4,44]. Moreover, multiple mechanisms may contribute to microgravity-induced osteoporosis. Studies have shown that non-coding RNAs [45,46], osteoblast exosomes, and epigenetic modifications play important roles in the regulation of osteoblast function and bone homeostasis in microgravity [47,48]. However, the exact molecular mechanism remains to be elucidated.

An increasing number of studies have corroborated the crucial role of melatonin in the prevention and treatment of osteoporosis. Recently, m6A modification has been identified as a key regulator of bone metabolism, with melatonin emerging as a potential modulator of biological processes by targeting m6A modification. This suggests that m6A may be implicated in the melatonin-regulated pathway of bone metabolism. However, there are few reports on the effect of melatonin targeting m6A modification on bone loss during mechanical unloading. Our study demonstrates, for the first time, that the melatonin/hnRNPA2B1 pathway regulates osteoblast differentiation under simulated microgravity.

Melatonin is an indole-like hormone secreted by the pineal gland in vertebrates. It is involved in a variety of physiological functions, including the regulation of sleep and circadian rhythms, the immune response, neuroprotection, etc. [49]. Previous studies have shown that melatonin can promote osteoblast differentiation and inhibit osteoclast activation. In vivo melatonin supplementation helps postmenopausal women and the elderly maintain bone mass and has the potential to prevent and control osteoporosis without side effects [27]. So far, there are no completely safe and effective comprehensive countermeasures, but aerospace medicine experts have proposed that melatonin may serve as a promising new drug to prevent bone loss during spaceflight [32]. However, the specific mechanism by which melatonin regulates microgravity-induced bone loss remains unknown.

m6A is the most common dynamic methylation modification located at the adenosine N6 site. As a highly conserved internal modification in eukaryotes, it plays a crucial role in both physiological and pathological conditions. m6A modification can regulate bone-related diseases, such as osteoporosis, osteoarthritis, and osteosarcoma [50]. It affects cell proliferation, differentiation, and apoptosis in bone-associated cells [51], such as bone marrow mesenchymal stem cells, osteoblasts, and osteoclasts [52], by regulating the expression of related genes and bone metabolic signaling pathways, including the PTH/Pth1r, PI3K-Akt, and Wnt/β-catenin pathways [50]. m6A plays a pivotal role in regulating almost every process of RNA processing and metabolism, including precursor RNA processing and RNA expression in the nucleus, as well as RNA translation and degradation in the cytoplasm. The regulation of m6A is carried out by methyltransferases, demethylases, and m6A readers. m6A readers are RNA-binding proteins that specifically recognize and bind to m6A-modified transcripts. They regulate mRNA splicing, folding, nuclear export, and translation and miRNA biosynthesis [40,53].

Recent studies have shown that hnRNPA2B1 can function as an m6A reader in numerous biological processes, including the regulation of mRNA maturation, transport and metabolism, as well as the processing of precursor miRNAs for translocation [54]. In this study, for the first time, we observed that hnRNPA2B1was downregulated both in vitro and in vivo during simulated microgravity. Furthermore, this study revealed that hnRNPA2B1 could promote osteoblast differentiation and that the overexpression of hnRNPA2B1 attenuated the suppression of osteoblast differentiation induced by simulated microgravity. This suggests that the inhibition of osteoblast differentiation under simulated microgravity may be partially associated with reduced hnRNPA2B1.

Melatonin has been shown to regulate a variety of signaling pathways and related functional proteins through m6A modifications in a number of biological processes, including spermatogenesis, testicular injury, and ovarian aging [55,56,57]. In this study, we found that melatonin could increase bone mass and improve the trabecular microarchitecture in HLU mice and could promote hnRNPA2B1 expression in osteoblasts under simulated microgravity. We further demonstrated that melatonin promoted osteoblast differentiation partly by targeting hnRNPA2B1. These results suggest that hnRNPA2B1 plays an important role in the regulation of osteoblast function in response to melatonin, and targeting the melatonin/hnRNPA2B1 pathway may, therefore, represent a potential protective strategy against microgravity-induced bone loss and osteoporosis.

However, this study has potential limitations. This study builds on previous research on the relationship between melatonin and osteoporosis. Different from the majority of direct mechanisms previously reported, the melatonin/hnRNPA2B1 pathway in this study represents an indirect mechanism. It is possible that the regulation of osteoblast differentiation by melatonin via m6A modification is not the most prominent mechanism through which melatonin exerts its effects. Furthermore, the mechanism by which hnRNPA2B1 is decreased in osteoblasts under simulated microgravity remains uncertain. Similarly, the mechanism by which melatonin regulates hnRNPA2B1 in osteoblasts under simulated microgravity is yet to be determined. In addition, the specific downstream mechanism by which hnRNPA2B1, an m6A reader, regulates osteoblast function in an m6A-dependent manner requires further investigation. Osteoblast differentiation factors include RUNX2, COL1A1, OCN, OSX, ALP, and others, of which RUNX2 is the most critical transcription factor in osteogenic differentiation. In this study, we selected RUNX2, COL1A1, and ALP, the three differentiation genes with more stable results, to analyze the osteoblast differentiation function. In addition, changes in OSX and OCN were observed, which are consistent with the findings of this study, but were not included in the results. The subsequent study of the m6A mechanism will include an analysis of OSX and OCN. Furthermore, melatonin has been shown to inhibit osteoclast activity [58,59]. It can inhibit microgravity-stimulated osteoclast activity by inhibiting RANKL gene expression and promoting calcitonin gene expression under simulated microgravity [32]. However, whether melatonin can inhibit osteoclast function via hnRNPA2B1 under simulated microgravity needs further investigation. Therefore, we will continue to investigate the effects of melatonin/hnRNPA2B1 on osteoclasts and bone remodeling under simulated microgravity to verify our hypothesis. In addition, the effect of melatonin/hnRNPA2B1 on bone homeostasis in osteoblast and osteoclast co-culture under simulated microgravity deserves further investigation. Despite these limitations, we believe that our study provides important new insights into the role of the melatonin/hnRNPA2B1 pathway in microgravity-induced bone loss and osteoporosis.

## 5. Conclusions

In conclusion, this study uncovered a microgravity-sensitive m6A reader, hnRNPA2B1, which plays an essential role in regulating osteoblast differentiation. Our findings show that hnRNPA2B1 effectively attenuated the restriction of simulated microgravity. Moreover, we demonstrated that melatonin could regulate osteoblast differentiation by alleviating the reduction in hnRNPA2B1 in osteoblasts caused by simulated microgravity. This research is the first to elucidate the function of the melatonin/hnRNPA2B1 axis in osteoblast differentiation under simulated microgravity. Targeting this axis may represent a prospective protective strategy against microgravity-induced bone loss and osteoporosis.

## Figures and Tables

**Table 1:** RNA Oligo sequences.

RNA Oligo	Sequences (5′-3′)
si-hnRNPA2B1 sense	5′-GGUGGCUUAAGCUUUGAAATT-3′
si-hnRNPA2B1 antisense	5′-UUUCAAAGCUUAAGCCACCTT-3′
si-NC sense	5′-UUCUCCGAACGUGUCACGUTT-3′
si-NC antisense	5′-ACGUGACACGUUCGGAGAATT-3′

**Table 2:** The sequence of primers.

Name of Primers	Sequences (5′-3′)
hnRNPA2B1-F	5′-GCGGAGGAAGAGGCGGTTAC-3′
hnRNPA2B1-R	5′-GTTAGAAGGCTGCTGGTTGTAGTTG-3′
Runx2-F	5′-GAACCAAGAAGGCACAGACAGA-3′
Runx2-R	5′-GGCGGGACACCTACTCTCATAC-3′
Col1a1-F	5′-GGCGGGACACCTACTCTCATAC-3′
Col1a1-R	5′-GGGACCCTTAGGCCATTGTGTA-3′
ALP-F	5′-GCAGTATGAATTGAATCGGAACAAC-3′
ALP-R	5′-ATGGCCTGGTCCATCTCCAC-3′
GAPDH-F	5′-TGTGTCCGTCGTGGATCTGA-3′
GAPDH-R	5′-TTGCTGTTGAAGTCGCAGGAG-3′

## Data Availability

The authors confirm that the data supporting the findings of this study are available within the article.

## Supplementary Materials

The following supporting information can be downloaded at https://www.mdpi.com/article/10.3390/cimb46090572/s1, Figure S1: Hindlimb unloading procedure used on mice; Figure S2: Melatonin improved cortical bone architecture in HLU mice.
