# Supplementary material for: Melatonin Regulates Osteoblast Differentiation through the m6A Reader hnRNPA2B1 under Simulated Microgravity
Source: Curr Issues Mol Biol. 2024 Sep 1;46(9):9624–38. doi: 10.3390/cimb46090572 (PMC11430354; doi:10.3390/cimb46090572)
Supplement: Supplementary file 1 [file cimb-46-00572-s001.zip › cimb-3132703-supplementary.pdf]

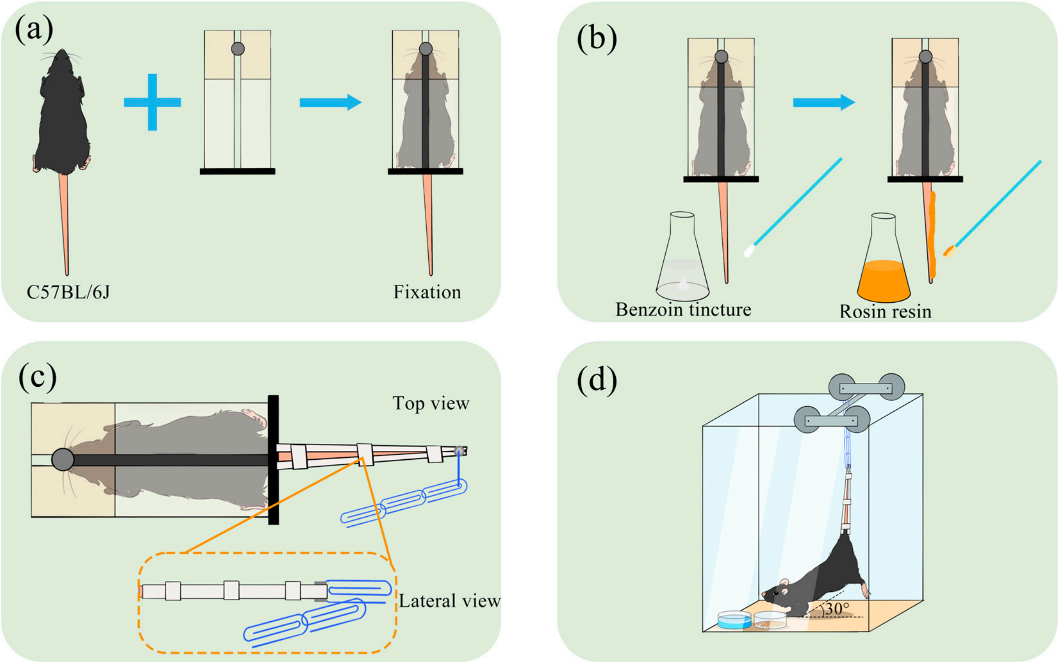

Figure S1. Hindlimb unloading procedure used on mice. (a) Fixation of mice. (b) A supersaturated solution of benzoin tincture (dissolved in anhydrous ethanol) was applied to tails of mice, which were naturally air dried and then coated with rosin resin. (c) Tape was applied to tails of mice, and paper clips were attached to the tape on tails. (d) Tails of mice were suspended from the crossbar of the special mouse cages, and the heights were adjusted so that mice's bodies were at 30° to the ground, with hindlimbs of mice suspended in the air.

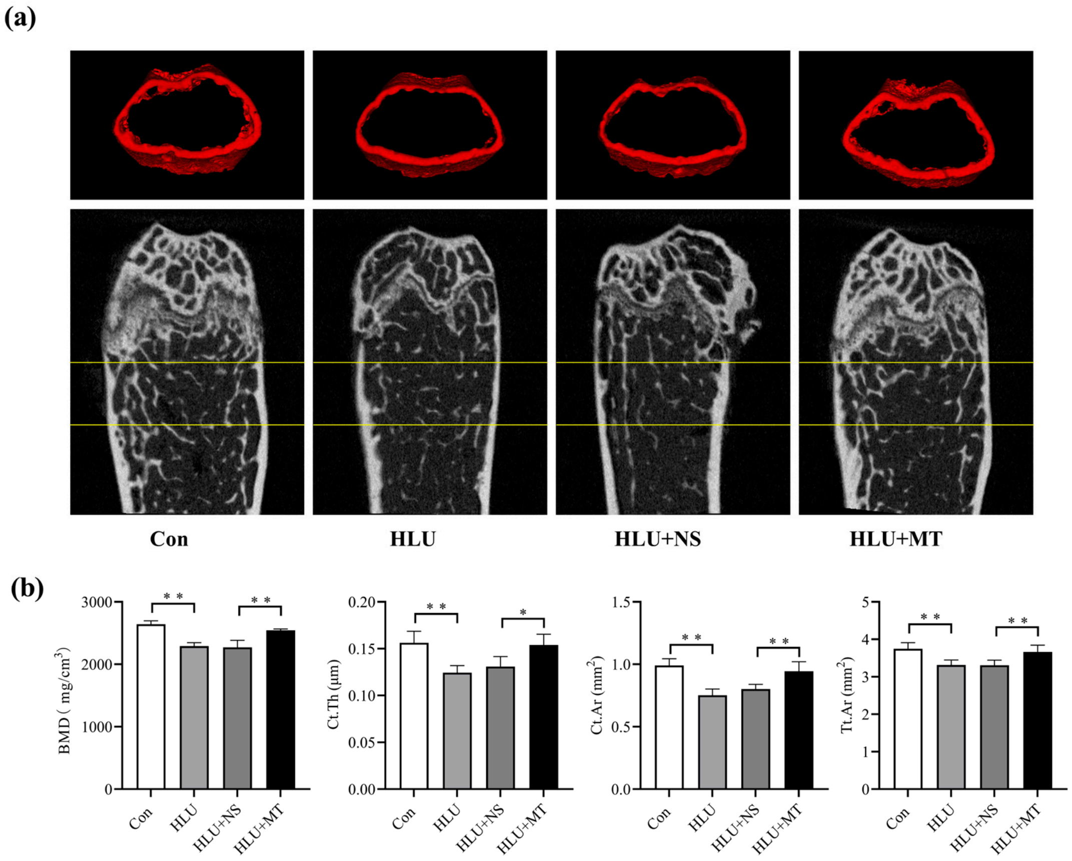

Figure S2. Melatonin improved cortical bone architecture in HLU mice. (a) Representative images of micro-CT and three-dimensional reconstruction of the distal femurs of mice in each group (N= 5). (b) Three-dimensional measurement of BMD, Ct. Th, Ct. Ar and Tt. Ar in the ROI region of the distal femurs of mice from each group (N=5). BMD: bone mineral density; Ct. Th: cortical bone thickness; Ct. Ar: cortical bone area; Tt. Ar: total cortical bone area.
